# A Highly Sensitive Multicommuted Flow Analysis Procedure for Photometric Determination of Molybdenum in Plant Materials without a Solvent Extraction Step

**DOI:** 10.1155/2017/9361460

**Published:** 2017-03-05

**Authors:** Felisberto G. Santos, Boaventura F. Reis

**Affiliations:** Centro de Energia Nuclear na Agricultura, Universidade de São Paulo, Av. Centenário, 303 São Dimas, 13400 970 Piracicaba, SP, Brazil

## Abstract

A highly sensitive analytical procedure for photometric determination of molybdenum in plant materials was developed and validated. This procedure is based on the reaction of Mo(V) with thiocyanate ions (SCN^−^) in acidic medium to form a compound that can be monitored at 474 nm and was implemented employing a multicommuted flow analysis setup. Photometric detection was performed using an LED-based photometer coupled to a flow cell with a long optical path length (200 mm) to achieve high sensitivity, allowing Mo(V) determination at a level of *μ*g L^−1^ without the use of an organic solvent extraction step. After optimization of operational conditions, samples of digested plant materials were analyzed employing the proposed procedure. The accuracy was assessed by comparing the obtained results with those of a reference method, with an agreement observed at 95% confidence level. In addition, a detection limit of 9.1 *μ*g L^−1^, a linear response (*r* = 0.9969) over the concentration range of 50–500 *μ*g L^−1^, generation of only 3.75 mL of waste per determination, and a sampling rate of 51 determinations per hour were achieved.

## 1. Introduction

Molybdenum is an essential micronutrient for plant growth, being a part of plant enzymes [1, 2]. The availability of Mo for plant growth is strongly affected by soil pH, with plants cultivated under Mo-deficient conditions showing growth impairment [3, 4]. Nevertheless, a narrow range of Mo concentrations is suitable for plants [5]. Consequently, the determination of Mo in plant materials allows foliar diagnosis, providing important information on the amount of fertilizer that should be used [6].

The molybdenum in plants has been determined using UV-vis spectrophotometry [7, 8], inductively coupled plasma mass spectrometry (ICP-MS) [9], flame atomic absorption spectrometry (FAAS) [10], and high-resolution continuum source flame atomic absorption spectrometry (HR-CS AAS) [11]. All these methods, except ICP-MS, require a preconcentration step to improve sensitivity, which has been implemented using solvent extraction. This additional step leads to more time-consuming analyses and, consequently, reduced throughput.

Among the above detection techniques, spectrophotometry presents several advantages, including its flexibility to be automated using flow injection analysis (FIA) [12, 13], methodology that has been widely used to develop analytical procedures [14, 15]. Spectrophotometry technique allows the use of cost-effective instrumentation, such as homemade photometers, assembled using light emitting diode (LED) and photodiode [16, 17]. However, the low sensitivity of this procedure presents a drawback, which is often overcome by including a solvent extraction step [7].

Bouger-Lambert-Beer's law implies that the sensitivity of a spectrophotometric analytical procedure is a function of the radiation beam path length in the sample [18, 19]. The availability of LED that emit highly intense radiation beam has allowed the use of flow cells with an optical path length of 100 mm, which has been exploited for developing highly sensitive photometric analytical procedures for chromium speciation and *N*-ureide determination in soybean tissues [20, 21].

Recently, a new flow cell design has been introduced, improving the radiation beam utilization and preventing also light scattering effect [18, 22]. Experimental results show that Bouger-Beer-Lambert's law is obeyed up to a flow cell length of 200 mm for LEDs emitting a radiation beam with a narrow opening angle [17]. This resource is exploited in the current study to develop a highly sensitive photometric procedure for the determination of Mo in plant materials, without the use of a solvent extraction step.

In acidic medium, Mo(V) reacts with thiocyanate ions to form a red-orange complex with an absorption maximum at 470 nm [7]. We exploit this phenomenon to develop an analytical procedure employing a homemade LED-based photometer comprising a blue LED (*λ* = 474 nm) as the radiation source and a 0PT301 photodetector. To improve sensitivity, the photometer was coupled to a flow cell with an optical path length of 200 mm. The analytical procedure was implemented using a multicommuted flow analysis process [23, 24], employing a flow system module with a syringe pump as the fluid propelling device.

## 2. Experimental

### 2.1. Reagents and Solutions

All solutions were prepared using purified water with an electric conductivity below 0.1 *μ*S cm^−1^. All chemical reagents were of analytical grade.

A 5.0% (w/v) potassium thiocyanate (KSCN) and 10% (w/v) tin(II) chloride dihydrate (SnCl_2_·2H_2_O) in 1.5 mol L^−1^ HCl solution were prepared by dissolving the solids in 6.25 mL of concentrated HCl (12 molL^−1^). After dissolution, the volume was made up to 50 mL with water. This solution was prepared daily.

A 1000 mg L^−1^ Fe(III) stock solution was prepared by dissolving 0.72 g of solid Fe(NO_3_)_3_·9H_2_O (Merck) in 5.0 mL of 12 mol L^−1^ HCl. After dissolution, the volume was made up to 100 mL with water.

A 1000 mg L^−1^ Mo(VI) stock solution was prepared by dissolving 0.1500 g of solid MoO_3_ (Merck) in 10 mL of 10 mol L^−1^ NaOH. After dissolution, 20 mL of a 5.0 mol L^−1^ HCl solution was added, and the volume was made up to 100 mL with water. From this stock solution a 10 mg L^−1^ Mo(VI) and 10 mg L^−1^ Fe(III) solution in 1.5 mol L^−1^ HCl was prepared to be used to prepare the working standards solutions with Mo(VI) concentrations ranging from 25 to 500 *μ*g L^−1^. These solutions were prepared daily, and the volumes were made up to 50 mL using a solution containing 1.5 mol L^−1^ HCl and 10 mg L^−1^ Fe(III).

### 2.2. Sample Preparation

Samples were prepared using a previously described protocol [11]. The dried and powdered plant material (5.0 g) was weighed into a porcelain crucible and ashed in a muffle furnace at 550°C for 5 h. After cooling to 25°C, 5.0 mL of 6 mol L^−1^ HCl solution was added, and the crucible was placed on a hotplate and heated to dryness. The residue was dissolved in 5.0 mL of 1.5 mol L^−1^ HCl solution and filtered through 0.45 *μ*m cellulose acetate paper. The solution was transferred into a volumetric flask, and the volume was made up to 25 mL using a 1.5 mol L^−1^ HCl and 10 mg L^−1^ Fe(III) solution [7].

### 2.3. Apparatus and Accessories

A microcomputer equipped with a PCL711 electronic interface card (Advantech) and running software written in Quick BASIC 4.5 was used in this study to control the flow system and to perform data acquisition. A motorized homemade syringe pump consisting of two 5.0 mL glass syringes and two three-way solenoid valves HP225T031 (NResearch), equipped with an electronic interface and controlled by the microcomputer, was used. The flow analysis manifold was constructed by assembling one three-way and two two-way solenoid valves HP225T031 and 161T011 (NResearch), one polyethylene reactor coil (100 cm long, 0.8 mm inner diameter), and Teflon-machined flow lines joints (one three-way and one four-way). A digital interface comprising two integrated circuits (ULN2803) was used to control the solenoid valves. The photometer was assembled using a 5.0 mm high-intensity emission LED with an emission maximum at 474 nm, a 0PT301 photodetector (Texas Instruments), a glass flow cell with an optical path length of 200 mm (1.2 mm inner diameter) [18], a BC547 transistor, and a 5.0 kΩ variable resistor. A stabilized power supply of (12 V, −12 V, and 0.5 A) was used to feed the photometer, and a stabilized power supply of 12 V (3 A) was used to feed the syringe pump and solenoid valves.

### 2.4. Description of the Flow Analysis Setup

**Figure 1:**
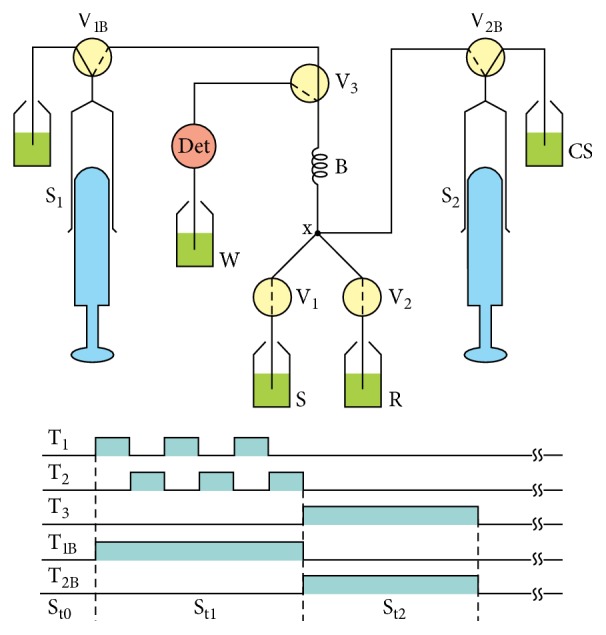
Diagram of the flow system manifold. S_1_ and S_2_ = syringes; V_1B_, V_2B_, and V_3_ = three-way solenoid valve; V_1_ and V_2_ = two-way solenoid; pumping flow rate at 50 *μ*Ls^−1^; A = sample; R = reagent (SnCl_2_ and KSCN); Cs = carrier solution (hydrochloric acid 1.5 mol L^−1^); B = reaction coil, 100 cm long and 0.8 mm inner diameter; Det = photometer, *λ* = 470 nm; W = waste; T_1_, T_2_, T_3_, T_1B_, and T_2B_ = switching time diagram for valves V_1_, V_2_, V_3_, V_1B_, and V_2B_, respectively. Dashed and solid lines in the valve symbols indicate the fluid pathway when the valves were switched on or off, respectively. Dashed lines in the symbols of valves V_1_ and V_2_ indicate that they are normally closed, therefore permitting fluid stream through them only when they are switched on. The shadow surfaces beneath the time lines (T_1_, T_2_, T_3_, T_1B_, and T_2B_) indicate that the respective device is switched on. St_0_ = stand by condition; St_1_ and St_2_ = sampling and signal reading steps, respectively.

The flow system setup used an automatic syringe pump to propel the fluid, assembled as shown in Figure 1. The forward and backward displacements of syringe pistons were performed using a direct current motor, as previously described [16]. In the configuration shown in Figure 1, the syringe pistons are in the middle position. When the microcomputer started the control software, the syringe piston position was identified by reading a signal generated by an end-course key [16]. The syringe pump motor rotated in a counterclockwise direction, displacing the syringe piston upward, thus emptying the syringe. After this step, the syringe pump was ready to begin the sampling step.

In the configuration of Figure 1, the flow system is at the stand by condition (St_0_) and to perform the sampling step (St_1_), the syringe pump motor rotated in a clockwise direction. Valve V_1B_ was maintained switched on, and solenoid valves V_1_ and V_2_ were sequentially switched on/off to insert slugs of sample (S) and reagent solution (R) into the reaction coil (B). This pattern was termed a sampling cycle and could be repeated several times to fill the reaction coil with a string of sample slugs followed by reagent solution slugs. The volume of each solution inserted into the reaction coil was a function of the pumping flow rate and time interval. After the required number of sampling cycles, the solenoid valves were switched off, and the reading step was performed. The motor of the syringe pump rotated clockwise, and solenoid valves V_3_ and V_2B_ were maintained in the on position. Under these conditions, the carrier solution displaced the sample zone from the coil through the photometer flow cell toward the waste outlet (W). The signal generated by the photometer was converted to digital and saved as an ASCII file to allow further processing. After this step, the system was able to start the next analytical sequence. The set of actions described in this paragraph is depicted in Table 1.

### 2.5. Calibration of the Photometer

The photometer, consisting of a photodiode, an LED, and a transistor, was coupled to a flow cell with an optical path length of 200 mm, which was molded and assembled as described elsewhere [18]. The flow cell possess a geometry that improves the radiation beam utilization. After propagating through the flow cell, the radiation beam was directed toward the observation windows of the photodetector (Det), which generated an electric potential difference that has a linear relationship with the final radiation beam intensity emitted by the LED.

When the control software was started, the user was prompted to calibrate the photometer. If calibration was needed, the microcomputer used the software calibration routine, driving the syringe module (Figure 1) to fill the flow cell with the carrier solution (Cs). Subsequently, the user was instructed to adjust the LED emission intensity to perform the full-scale measurement (Ms = 2000 mV). Subsequently, the flow cell was filled with an intensely colored solution, obtained by mixing equal volumes of a 15 mgL^−1^ Fe(III) solution with the previously mentioned thiocyanate solution, forming a compound that absorbed radiation with maximum at 480 nm. The signal generated was denoted as diffuse measurement (Dm). The Ms and Dm measurement were saved for the absorbance calculation:

(1) $$\begin{array}{c} Absorbance=\log\left[\;\frac{\left(Ms-Dm\right)}{\left(S-Dm\right)}\;\right], \end{array}$$

where Ms is full-scale measurement; Dm is diffuse measurement; S is signal generated for the flow cell filled with a light absorbing solution.

The assays showed that when Ms was kept close to 2000 mV, Dm maintained a value of 27.9 mV. When assays were performed using Fe(III) solutions with concentrations higher than 15 mgL^−1^, the results were practically identical to the referred value of the diffuse measurement, thus indicating that this signal is not dependent on the solution inside the flow cell.

Previous work proved that the above equation effectively describes the response of the LED-based photometer [18], and therefore, this strategy was adopted in the current work. The photometer was calibrated 20 min after being switched on, while the Dm assay was carried out once a week.

## 3. Results and Discussion

### 3.1. Effect of Reducing Reagent Concentration

The detection procedure was based on the reaction of Mo(V) with thiocyanate; however, the analyte is present as Mo(VI) in the sample solution. Usually, Sn(II) chloride has been used as a reducing reagent to convert Mo(VI) to Mo(V), which then reacts with thiocyanate to form a colored complex that can be monitored at 470 nm [7, 28, 29]. Since the reduction of Mo(VI) is essential for the formation of the colored compound, the effect of Sn(II) concentration was the first parameter evaluated. Assays were performed by varying the Sn(II) solution (prepared in 1.5 mol L^−1^ HCl) concentration from 0.04 to 0.8 mol L^−1^ and using blank and 150 *μ*g L^−1^ Mo(VI) standard solutions as models.

**Figure 2:**
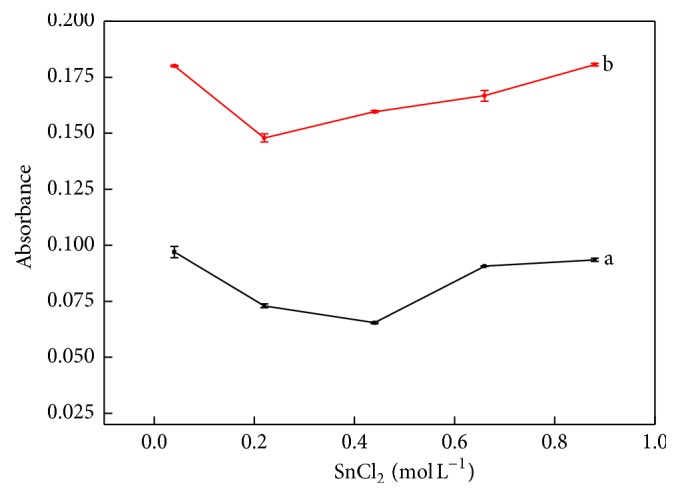
Effect of the concentration of the reducing agent. Curves a and b correspond to the blank and a 150 *μ*gL^−1^ Mo (VI) standard solution, respectively. Experimental conditions: 0.5 mol L^−1^ SCN^−^; seven sampling cycles; time intervals to insert sample and SCN^−^ solution 2.0 and 1.0 s, respectively; pumping rate at 50 *μ*Ls^−1^.

The results displayed in Figure 2 show that the magnitude of blank measurements is minimal for a Sn(II) concentration of 0.44 mol L^−1^, tending to be constant for concentrations above 0.75 mol L^−1^.

The curve obtained for the 150 *μ*g L^−1^ Mo(VI) standard solution shows a different behavior, displaying a slight increase in signal magnitude for Sn(II) concentrations between 0.2 and 0.9 mol L^−1^. The largest difference between the two curves was observed for a concentration of 0.44 mol L^−1^, which was selected for further experiments.

### 3.2. Effect of Acid Concentration

The reaction of Mo(V) with SCN^−^ occurs in acidic medium [28], and hence, to evaluate the effect of acidity, a set of assays was performed using blank and 100 *μ*g L^−1^ Mo(VI) solutions, prepared in HCl medium with concentrations ranging from 1.0 to 2.5 mol L^−1^. Taking the absorbance of blank and Mo(VI) solutions as a function of HCl concentration, we observed that absorbance increased linearly in both cases, as shown by the following equations: absorbance (blank) = 0.0117*x* + 0.0595 (*r* = 0.9687); absorbance (Mo) = 0.0163*x* + 0.0935 (*r* = 0.9897). The slope observed for the molybdenum solution is 30% higher than that of the blank, indicating that HCl concentration affects signal magnitude. Considering these results, a 1.5 mol L^−1^ HCl solution was selected for further assays.

### 3.3. Effect of Thiocyanate Concentration

**Figure 3:**
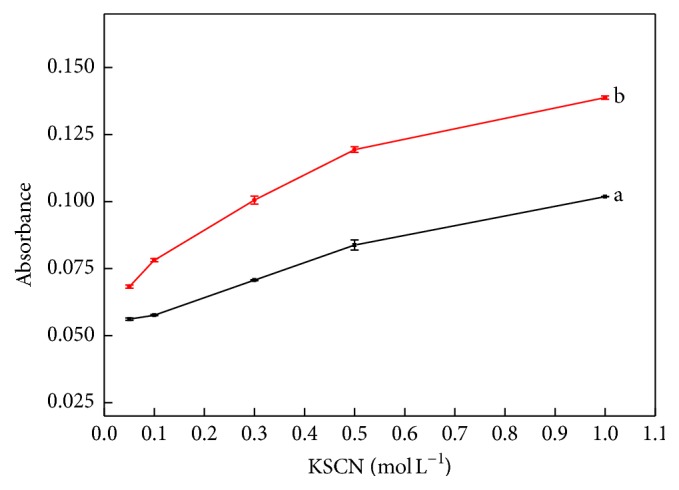
Effect of the potassium thiocyanate concentration. Curves a and b refer to blank and Mo (VI) standard solution (100 *μ*gL^−1^). Experimental conditions: 0.44 mol L^−1^ SnCl_2_; blank and Mo (VI) standard solution in 1.5 mol L^−1^ HC medium; 7 sampling cycles; sampling time intervals of 2.0 and 1.0 s for insertion sample and SCN^−^ solution, respectively; pumping rate at 50 *μ*Ls^−1^.

The results presented in previous sections were obtained using a 0.5 mol L^−1^ thiocyanate solution. To determine the optimum concentration, assays were performed using KSCN solutions with concentrations between 0.05 and 1.02 mol L^−1^ and results are shown in Figure 3.

The curves displayed in Figure 3 show that the absorbance obtained for both solutions increased up to a KSCN concentration of 0.5 mol L^−1^. For higher concentrations, the curves became parallel, indicating an increase of blank measurement absorption without a gain in sensitivity. Based on these results, a KSCN concentration of 0.5 mol L^−1^ was selected.

### 3.4. Effect of Fe Concentration

**Figure 4:**
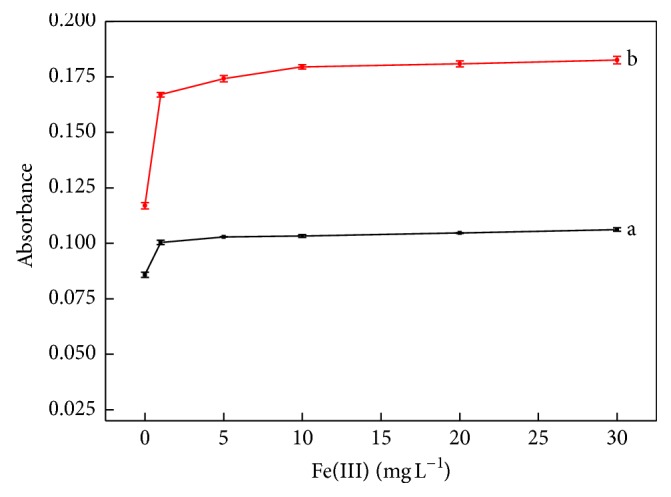
Effect of the iron (III) concentration on sensitivity. Curves a and b are related with blank and a 150 *μ*g L^−1^ Mo(VI) standard solution, respectively. Experimental conditions: 0.44 mol L^−1^ SnCl_2_; blank and Mo (VI) standard solution in 1.5 mol L^−1^ HC medium; 7 sampling cycles; sampling time intervals of 2.0 and 1.0 s for insertion sample and SCN^−^ solution, respectively; pumping rate at 50 *μ*Ls^−1^.

The use of Fe(III) for improving sensitivity is a common practice in the thiocyanate-based spectrophotometric determination of Mo using Sn(II) as the reducing reagent [28, 29]. To evaluate the effect of Fe(III) concentration on sensitivity, a set of assays was performed. Blank and 150 *μ*g L^−1^ Mo(VI) solutions were prepared with Fe(III) concentrations varying from 0 to 30 mg L^−1^, with the results shown in Figure 4.

Analysis of the curves in Figure 4 reveals that both blank and Mo solutions showed significant absorption increases as the concentration of Fe(III) changed from 0 to 2.0 mg L^−1^, being more significant for the Mo(VI) standard solution. Since both curves showed a practically invariant behavior for Fe(III) concentrations above 10 mg L^−1^, this concentration was selected for the preparation of standard solutions and samples.

### 3.5. Effect of the Sample Slug Volume

**Figure 5:**
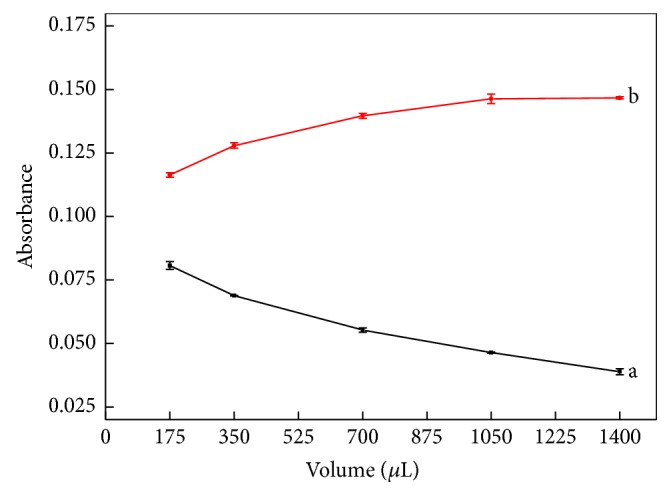
Effect of the sample slug volume. Curves a and b correspond to blank and the 150 *μ*g L^−1^ Mo(VI) standard solution, respectively.

In multicommuted flow analysis approach, sample and reagent solution slugs are sequentially inserted into the reaction coil [30], with the mixing required for reaction development occurring by dispersion through solution interfaces. Consequently, an appropriate ratio between the sample and reagent solution slug volumes is essential for proper reaction development. Furthermore, efficient mixing conditions improve both sensitivity and measurement precision. For this reason, the sample slug volume per sampling cycle was varied from 25 to 200 *μ*L by changing the time interval of keeping valve V_1_ (Figure 1) switched on from 0.5 and 4.0 s. The reagent solution slug volume used in each sampling cycle equaled 50 *μ*L. The sampling step (Table 1) comprised seven sampling cycles, and, therefore, the volume of the sample zone was varied from 525 to 1750 *μ*L, yielding the results shown in Figure 5.

The curves displayed in Figure 5 show that the analytical signal magnitude increased up to a sample slug volume (per sampling cycle) of 150 *μ*L, with no further increase thereafter. This result is expected, considering that the volume of the sample zone 1400 *μ*L (sample 1050 *μ*L, reagent solution 350 *μ*L) is higher than the volume of the analytical path, which comprised the volumes of the reaction coil (500 *μ*L) and flow cell (210 *μ*L), thereby minimizing the dispersion effect. The results of blank measurements showed a constant decrease of signal magnitude, which was also expected, as increasing the volume of the sample slug causes a dilution of the reagent solution. Based on these results, a sample slug volume of 150 *μ*L per sampling cycle was selected.

### 3.6. Evaluation of Potential Interferences

The chemical elements W, Ti, Re, U, V, Co, Cu, and Bi can react with thiocyanate [28, 29], possibly causing interference. In plant material, the concentrations of U, W, Ti, Re, V, and Bi are usually very low; therefore, we focused on Co and Cu, because their concentrations are usually high enough to cause interference [28, 31]. Assays were performed by adding a certain amount of the potential interferent to the standard Mo(VI) solution and comparing the measurement results with those achieved without the assayed interferent and results are shown in Table 2.

Usually, a measurement variation of ±5% is taken as an interference criterion [22]. According to this criterion, the results in Table 2 indicate no significant interference. The Co(II) and Cu(II) concentrations used in these assays are higher than those usually found in plant materials. Thus, no interference is expected for the analysis of real samples.

### 3.7. Application and Performance Comparison

Intending to access the overall response of both equipment setup and analytical procedure, assays were performed using set of Mo(VI) standard solutions. By processing the results we achieved a linear response (*r* = 0.9969) for concentration within the range of 50 to 500 *μ*g L^−1^ Mo(VI), a detection limit of 9.1 *μ*g L^−1^ Mo(VI) (*n* = 11, 3*α* criterion), and a 1.07% relative standard deviation using a 250 *μ*g L^−1^ Mo(VI) standard solution.

To assess the usefulness of the proposed procedure, its accuracy was evaluated by the analysis of two sets of samples mineralized as described in Section 2.2. A set of soybean samples was also analyzed by inductively coupled plasma optical emission (ICP-OES) [32], while another set was analyzed using also the spiked recovery tests and results are shown in Tables 3 and 4.

Application of the paired *t*-test to the results in Table 3, considering a 95% confidence level and three degrees of freedom, results in a value of 0.83, which is much lower than the reference value (3.18). Thus, no significant difference exists between the results of these two methods.

Molybdenum concentrations in the other sample set were at the *μ*gL^−1^ level, which is beyond the working range of ICP-OES. Therefore, the accuracy assessment was performed through the spike recovery tests. The results shown in Table 4 indicate recoveries between 82 and 113%, being acceptable for samples with low analyte concentrations [10, 33–35].

To compare the performance of the proposed procedure with those of existing procedures for spectrophotometric Mo determination, the main analytical parameters reported for these methods are presented in Table 5.

The results show that the proposed procedure presents a wide analytical concentration range and a higher sampling rate than those achieved previously, while the reagent consumption and waste generation are in favor of the current work. Notably, the superiority of the proposed method was achieved without the use of a solvent extraction step, which is often used to improve sensitivity [7, 8].

## 4. Conclusions

The proposed procedure allows highly sensitive analysis without the use of an organic solvent, achieved through the use of an LED-based photometer equipped with a flow cell having a long optical path length. A multicommuted flow analysis module allowed analysis of samples with a wide Mo concentration range without changing the flow system.

The proposed methodology achieved enhanced performance, for example, high throughput, low reagent consumption, and reduced volume of generated waste. Thus, according to the analytical green chemistry guidelines [36], this procedure can be considered a clean method. The flow system module, LED-based photometer, and proposed analytical procedure constitute a cost-effective approach for Mo determination at *μ*g L^−1^ concentrations as a better alternative to existing procedures.

## Figures and Tables

**Table 1:** Sequence of events related to an analytical run.

Step	Event	Rotation	V_1B_	V_2B_	V_1_	V_2_	V_3_	Time (s)
1	Filling reagent channel	L	1	0	0	1	0	4
2	Washing analytical path	R	0	1	0	0	1	18
3	Photometer calibration	S	0	0	0	0	0	—
4	Filling sample channel	L	1	0	1	0	0	5
5	Filling syringe with carrier fluid	L	0	1	0	0	1	18
6	Washing sample channel	R	1	0	1	0	0	18
7	Sampling step	L	1	0	1	1	0	10
8	Signals reading step	R	0	1	0	0	1	—

The L, R, and S in the rotation column = motor rotation direction for left, right, and stop, respectively. V_1B_, V_2B_, V_1_, V_2_, V_3_ = solenoid valves. Numbers 0 and 1 indicated that related valve was switched off or on, respectively. The numbers in the last column at right are the selected time intervals.

**Table 2:** Effect of the potential interferents on the analytical signal.

Standard Mo(VI) (mgL^−1^)	Addition (mgL^−1^)	Signal (Abs)	Addition (mgL^−1^)	Signal (Abs)
	Cu		Co	
0.5	—	0.4918 ± 0.0017	—	0.4737 ± 0.0032
0.5	0.5	0.4843 ± 0.0030	0.5	0.4668 ± 0.0037
0.5	2.5	0.4755 ± 0.0062	1.0	0.4730 ± 0.0042
0.5	5.0	0.4855 ± 0.0042	2.0	0.4678 ± 0.0033
0.5	10	0.4982 ± 0.0032	5.0	0.4632 ± 0.0063

**Table 3:** Results comparison.

Sample	Proposed procedure Mg L^−1^	Reference method (ICP OES) mg L^−1^
1	2.27 ± 0.01	1.75 ± 0.01
2	7.25 ± 0.01	6.88 ± 0.02
3	19.01 ± 0.45	20.39 ± 0.07
4	13.07 ± 0.07	9.20 ± 0.01

Results are average of three consecutive measurements.

**Table 4:** Results of spiked recovery tests.

Sample	Analyte concentration (*μ*g L^−1^)	Spiked concentration (*μ*g L^−1^)	Found concentration (*μ*g L^−1^)	Recovery%
1	74.3 ± 1.0	80	157.3 ± 0.6	103.7
2	94.3 ± 2.5	80	185.2 ± 3.6	113.5
3	73.1 ± 1.9	80	146.8 ± 2.3	92.2
4	73.9 ± 3.1	80	140.1 ± 2.6	82.7
5	73.9 ± 3.1	75	135.8 ± 1.6	82.5
6	73.1 ± 1.9	75	134.7 ± 3.4	82.1
7	74.3 ± 1.0	75	136.0 ± 1.3	82.2

Results are average of three consecutive measurements.

**Table 5:** Performance comparison.

Approach	Parameters						Ref.
	Linear range (*μ*gL^−1^)	Detection limit (*μ*gL^−1^)	Sampling rate (h^−1^)	Consumption (mg)^*∗*^	Waste (mL)^*∗*^	Sol.^a^ (*μ*L^−1^)	
MCFA	50–500	9.1	51	17.5^b^	3.7	—	This work
				35^c^			
MCFA	25–150	4.6	25	30^b^	2.03	200	[7]
				30^c^			
FIA	50–1000	—	30	64^b^	14.6	1400	[8]
				64^c^			
FIA	0–100	0.6	50	24^d^	4.9	—	[25]
SIA	5–80	2.4	25	—	—	333	[26]
FIA	1–20	0.5	15	—	8	—	[27]

^*∗*^Reagent consumption and waste generation per determination; ^a^organic solvent, consumption per determination; ^b^thiocyanate; ^c^tin(II) chloride; ^d^potassium iodide.
